# Full-waveform inversion imaging of the human brain

**DOI:** 10.1038/s41746-020-0240-8

**Published:** 2020-03-06

**Authors:** Lluís Guasch, Oscar Calderón Agudo, Meng-Xing Tang, Parashkev Nachev, Michael Warner

**Affiliations:** 1 Department of Earth Science and Engineering, Imperial College London, London, SW7 2AZ UK; 2 Department of Bioengineering, Imperial College London, London, SW7 2AZ UK; 3 Institute of Neurology, University College London, 33 Queen Square, London, WC1N 3BG UK

**Keywords:** Ultrasonography, Brain imaging, Three-dimensional imaging, Tomography, Acoustics

## Abstract

Magnetic resonance imaging and X-ray computed tomography provide the two principal methods available for imaging the brain at high spatial resolution, but these methods are not easily portable and cannot be applied safely to all patients. Ultrasound imaging is portable and universally safe, but existing modalities cannot image usefully inside the adult human skull. We use in silico simulations to demonstrate that full-waveform inversion, a computational technique originally developed in geophysics, is able to generate accurate three-dimensional images of the brain with sub-millimetre resolution. This approach overcomes the familiar problems of conventional ultrasound neuroimaging by using the following: transcranial ultrasound that is not obscured by strong reflections from the skull, low frequencies that are readily transmitted with good signal-to-noise ratio, an accurate wave equation that properly accounts for the physics of wave propagation, and adaptive waveform inversion that is able to create an accurate model of the skull that then compensates properly for wavefront distortion. Laboratory ultrasound data, using ex vivo human skulls and in vivo transcranial signals, demonstrate that our computational experiments mimic the penetration and signal-to-noise ratios expected in clinical applications. This form of non-invasive neuroimaging has the potential for the rapid diagnosis of stroke and head trauma, and for the provision of routine monitoring of a wide range of neurological conditions.

## Introduction

No universally applicable means of imaging the living human brain at high anatomical resolution exists. The modality with the best spatial resolution and tissue contrast, magnetic resonance imaging (MRI), is contraindicated where the presence of magnetic foreign bodies cannot be excluded, and is impractical with claustrophobic, uncooperative, or severely obese patients. Its nearest rival, X-ray computed tomography (CT), involves exposure to harmful ionising radiation. Both require large, expensive, immobile, high-power instruments that are near-impossible to deploy outside specialised environments. The clinical consequences of this are high symptom-to-image times, long inter-scan intervals during serial imaging, and constraints on the range of patients that can be imaged successfully.

Pre-eminent among the many neurological disorders, where patient outcomes are degraded by these restrictions, is stroke: the second most common cause of death worldwide, and the dominant cause of acquired adult neurological disability^1^. Treatment decisions are here critically guided by neuroimaging, ideally performed immediately after symptom onset. Delays of the order of minutes have substantial impact on outcomes, yet the necessity to treat patients only after transport to hospital routinely introduces delays of an hour or more^2^. Accelerating the treatment of stroke by enabling neuroimaging and treatment to be performed at the point of first contact would thus have large population-level impacts on survival and disability. Analogous arguments can be made for improved rapid medical imaging in head trauma, and in routine intraoperative, post-operative, and preventative neurological monitoring, with the potential to impact large numbers of patients worldwide.

We provide in silico proof-of-principle, supported by ex vivo and in vivo laboratory measurements, that the combination of transmitted transcranial ultrasound tomography with a computationally intensive technique originally developed to image the interior of the Earth, can address these clinical needs by providing portable three-dimensional (3D) quantitative imaging that is less-expensive, faster, and more easily applicable than MRI, and that is safer and has better soft-tissue contrast than CT. This approach results in a three-dimensional, sub-millimetre-resolution, quantitative model of acoustic wave speed within the brain and surrounding tissue, which is capable of distinguishing most of the structures and pathologies to which MRI is sensitive. The combined findings of our in silico and laboratory experiments demonstrate that recording transcranial ultrasound data are feasible, recorded signal-to-noise levels are high, and the data contain information that is sufficient both to build an accurate model of the skull and to reconstruct brain properties. To achieve this, we use two closely related techniques: adaptive waveform inversion (AWI)^3^ to build an accurate skull model, and FWI^4^ to extract brain properties at high resolution.

Conventional medical ultrasound is fast, safe, portable, and cheap, but is unable to image the adult human brain at high resolution within the skull; the main reasons for this are well understood^5–8^:

1. In both conventional pulse-echo B-mode sonography^9^ and time-of-flight ultrasound CT^10^, high frequencies are required in order to obtain high spatial resolution. Scattering and anelastic losses occur within the skull and the brain, and these increase with frequency. At the frequencies used by conventional ultrasound modalities, these signal losses prevent successful imaging of intracranial soft tissue.
2. The contrast in wave speed between the skull and soft tissues, and between the skull and air-and-fluid-filled cavities within it, produces significant refraction, diffraction, and reverberation of ultrasound energy, as it is transmitted through the skull. This significantly distorts and complicates the consequent wavefront, leading to strong aberrations in both phase and amplitude, and to significant spatial and directional variation in the waveform of the transmitted pulse. It is not currently possible to correct these effects with sufficient accuracy using conventional modalities.
3. In pulse-echo sonography, back-scattered reflections are used to generate the image. The bones of the skull differ significantly in wave speed and density from those of surrounding soft tissue. Consequently, the skull generates strong reflections and multiple scattering, and these high-amplitude signals overlie, interfere with and obscure the much-weaker reflections produced by the small impedance contrasts that occur within soft tissue in the brain, leading to low signal and high source-generated noise in intracranial pulse-echo images.
4. Time-of-flight tomography uses a short-wavelength approximation, basing its analysis on the simplified physics of ray theory in which the effects of transmission through a heterogeneous medium are represented by a simple change in travel time. For a finite wavelength, wave transmitted through a medium that is heterogeneous on many scales, such delay times are only sensitive to the properties of the medium averaged over the dimensions of the first Fresnel zone^11^. Consequently, time-of-flight tomography is unable to resolve structure below this scale, and so lacks acceptable resolution at the low frequencies that can be recorded using transcranial ultrasound.

One possible way around these problems is to use natural openings in the skull as acoustic windows, but this approach severely reduces illumination^7^; it is typically limited to neonates through an open fontanelle^12^. In principle, it is also possible to remove, or thin, portions of the skull in order to record conventional high-frequency pulse-echo B-mode reflection images without strong bone reflections, absorption, or distortion. This method has produced promising results in rodents, generating functional ultrasound images that can capture transient changes in blood volume related to brain activity^13,14^, but this invasive approach has obvious limitations in clinical practice.

In this paper, we present the neurological application of full-waveform inversion (FWI)^4^, an imaging method first applied widely in geophysics^15^. FWI is a computationally intensive technique that has been developed to a high level of sophistication by the petroleum industry to image hydrocarbon reservoirs within the Earth^16,17^. The spatial resolution that can be obtained using this technique is much finer than that of time-of-flight tomography. FWI achieves this improved resolution through a combination of characteristics^18^, of which the most important is that it uses a more-complete description of the physics of wave propagation in heterogeneous media that takes proper account of the finite wavelength of transmitted waves. This description, which involves the full numerical solution of the wave equation, is able to model accurately the effects of sub-Fresnel zone heterogeneity and multiple scattering on the wavefield. FWI combines this more-accurate description of the physics with an appropriate non-linear inversion scheme, and a suitable data acquisition geometry, so that it is able to recover fine-scale heterogeneity throughout the model. Adaptive waveform inversion (AWI)^3,19^ is a modification of FWI that is better able to begin from a poor starting model; here we use it as a preconditioner for FWI so that inversion can begin successfully without any a priori information about the skull.

**Fig. 1:**
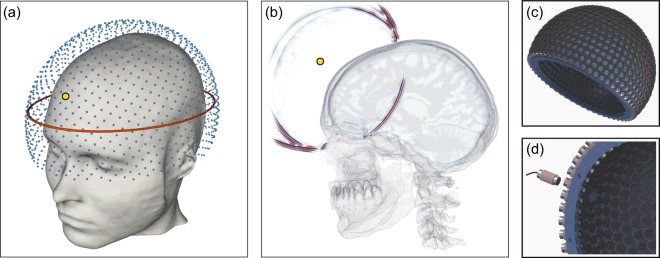
Experimental geometry. **a** Three-dimensional array of transducers used for data generation and subsequent inversion. Each transducer acts as both a source and a receiver. The red ellipse shows the location of the two-dimensional array used to generate the data for Fig. 2 and 4. **b** A snapshot in time of the wavefield generated by a source transducer located at the position indicated by the small yellow circle, computed via numerical solution of the 3D acoustic wave equation. The wavefield is dominated by strong reflections from the skull, and by intracranial transmitted energy travelling across the brain; Supplementary Video 1 shows the full wavefield propagating in time. **c** Prototype helmet containing 1024 transducers held rigidly in a 3D-printed framework. In the prototype device, the framework is customised to provide an accurate fit to an individual subject, and filled with water. In portable clinical devices, the sensors move radially, and contact the patient via sonographic gel. **d** Close up of sensor connections in the prototype.

Figure 1 outlines the geometry of the method. Low-frequency ultrasound data are recorded at all available azimuths by surrounding the head with ultrasound transducers in three dimensions. Each transducer is activated separately in turn as a source of ultrasound energy, and the signals that it generates are recorded by every other transducer. Allowing for reverberations to die away after each activation, it takes ~2 seconds in total to acquire a full data set for all 1024 sources each recorded on 1024 receivers. FWI uses predominantly transcranial transmitted energy recorded on the side of the head opposite to the source transducer, but it also extracts information from all other parts of the recorded wavefield including reflections, diffractions, multiple scattering, and guided waves that arrive at any angle at any of the transducers. Unlike conventional ultrasound imaging, FWI does not use focused transducers, focusing arrays or any type of beam forming, either in the experimental configuration or in the computer subsequently

The paper is organised as follows: we explore our proposed methodology using in silico simulations, and present in vivo and ex vivo laboratory results that support our assumptions. We begin by demonstrating the improvement in resolution provided by FWI in even the simplest case when the model is two-dimensional and the skull has been removed. We follow this by exploring what FWI is able to achieve in ideal circumstances for the intact adult human head in three dimensions; this result demonstrates the resolution and tissue contrast potentially achievable in a clinical setting. We follow this by demonstrating that a combination of AWI and FWI can begin from the simplest of starting models, and we demonstrate the importance of full 3D data acquisition and inversion. We present laboratory results, using direct observations of in vivo and ex vivo transcranial ultrasound, to demonstrate that good signal penetration and high signal-to-noise levels are readily achievable. We provide an example of the clinical relevance of our approach by demonstrating the accurate recovery of an intracranial haemorrhage, and discuss clinical applications to stroke and other pathologies. We conclude with an outline of our methodology and algorithms. In Supplementary Information, we discuss how our idealised in silico simulations and conclusions are likely to compare with those obtainable from real-world in vivo observations involving anelastic absorption and noise; we review spatial resolution in pulse-echo, time-of-flight tomography, and FWI methodologies; and we explore some of the practicalities and speed of implementation in likely clinical settings.

## Results

### Resolution with the skull removed

**Fig. 2:**
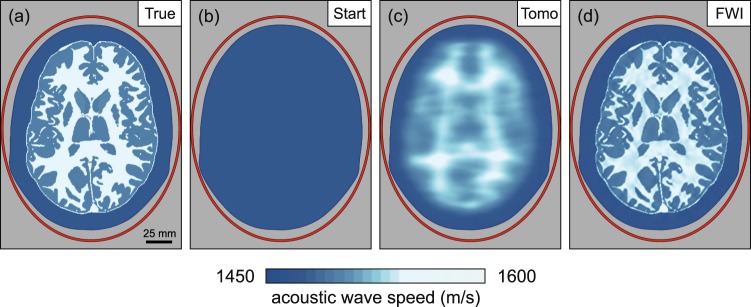
Inversion of data from a brain outside the skull. **a** A two-dimensional model of acoustic wave speed in the naked brain without the skull. The red ellipse shows the transducer positions; the grey region is masked and held fixed during the inversions. **b** Homogeneous model used to begin inversion. **c** Result of ultrasound computer tomography. The resultant model is accurate but has poor spatial resolution. **d** Result of ultrasound full-waveform inversion. The resultant model is now both accurate and spatially well resolved.

Ray-based time-of-flight tomography and wave equation-based FWI both represent forms of transmission tomography. Figure 2 demonstrates the difference between these two techniques using a simple two-dimensional model of the naked brain without the complicating effects of the skull. Using the model from Fig. 2a, and solving a numerical wave equation, a synthetic data set was generated for transducers located around the brain. Using the homogeneous starting model shown in Fig. 2b, this data set was inverted using both time-of-flight tomography and FWI, to recover the models shown in Fig. 2c, d. Time-of-flight tomography seeks to find the best-fitting model by using geometric ray theory to predict delay times for every source-receiver pair in the data set, whereas FWI seeks to solve the same problem by using the wave equation to predict the detailed variation of acoustic pressure with time recoded at every receiver for every source.

For this numerical experiment, the wavelength in soft tissue of the dominant frequency in the insonifying pulse was ~3.75 mm, the shortest wavelength was ~1.75 mm, and the minimum diameter of the Fresnel zone for signals that travelled across the model was > 20 mm. For conventional pulse-echo B-mode sonography, spatial resolution depends upon several factors^20^, but when imaging through relatively homogeneous soft tissues with an appropriate transducer configuration and a short pulse length of two cycles, the typical resolution is of the order of the insonifying wavelength at the dominant frequency, or ~3.75 mm in the present context. For wave equation-based transmission tomographic methods, such as FWI, the achievable resolution is of the order of half the wavelength of the highest available frequency^16,21^, or ~0.88 mm in the present context. For FWI, neither the pulse duration nor beam width directly affect spatial resolution.

In contrast, well-established theory^11^ and numerical experiments^22^ show that the maximum spatial resolution that can be achieved, in the far field using ray-based time-of-flight tomography, is of the order of the diameter of the first Fresnel zone. Thus, we would expect that an FWI model would be ~ 20 times better resolved in linear dimensions than the equivalent time-of-flight model. Figure 2 illustrates this behaviour directly. Both methods recover models that are accurate in their locally averaged properties, but the time-of-flight model has only centimetre-scale spatial resolution, whereas the FWI model has millimetre resolution. Note that, in this simple example, the difference in resolution between the two techniques is not related to the presence of the skull, nor to differences in the optimisation scheme—both methods used non-linear least-squares inversion applied to the same input data. A more-detailed discussion of the resolution achievable by pulse-echo sonography, time-of-flight tomography, and FWI is provided within the Supplementary Information.

In the absence of the skull, conventional high-frequency pulse-echo sonography would of course be able to recover an accurate and well-resolved image of the naked brain. However, if the skull interposes, then pulse-echo fails entirely to image the brain inside the skull because brain reflections are then significantly distorted, absorbed, and scattered by the skull. Similarly, time-of-flight tomography for the brain inside the intact human skull fails because, at the low frequencies that can be transmitted across the head with acceptable signal-to-noise ratios, spatial resolution is insufficient. FWI does not suffer from either of these problems; it properly accounts for the distorting effects of the skull, and it achieves good spatial resolution even at the low frequencies that can be recorded after transmission through the skull.

### Three-dimensional full-waveform imaging through the skull

FWI has obvious advantages for brain imaging; it does though have two complications of its own: the computational effort required to extract the image from the data in three dimensions is significant, and the method requires a reasonably good starting model in order to proceed to the correct final model.

The former requirement has, until recently, limited the applicability of medical FWI to problems that can be usefully solved in two dimensions^23^, and the skull is not even approximately two-dimensional. The advent of large parallel multi-core multi-node compute clusters, of on-demand parallel cloud computing, and of large-memory graphics processing unit (GPU) systems, coupled with improved FWI software and the use of pre-trained supervised deep-learning to accelerate the process, are reducing the computational demands of this method; runtimes and costs continue to reduce year-on-year.

The requirement for a good starting model is straightforward for the soft tissue of the brain where a homogeneous starting model is adequate. For the bones of the skull, however, additional care is required. In this section, we assume that the skull model is known a priori. In the following section, we demonstrate that AWI is capable of building an accurate model of the skull entirely from the ultrasound data without a priori knowledge, and that following this with FWI then recovers a fully resolved model of the brain.

**Fig. 3:**
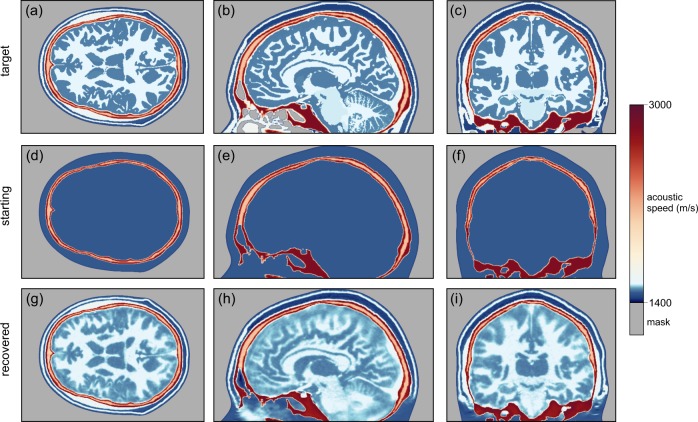
Models of acoustic wave speed. Transverse (left), sagittal (centre), and coronal (right) sections through the true (top), starting (middle), and recovered (bottom) models. Both the wavefield modelling and waveform inversion are performed in three dimensions. The starting model includes the true model of the skull, but is otherwise homogeneous.

Figure 3 shows transverse, sagittal, and coronal sections through a three-dimensional target model of wave speed, a starting model containing the true skull but otherwise homogeneous, and the model reconstructed using FWI applied to sub-MHz ultrasound data generated by the target model. Supplementary Videos 2, 3, and 4 show the true, starting, and reconstructed models in three dimensions. The colour scale shown in Fig. 3 is designed to highlight heterogeneity within both soft and hard tissues.

The bones of most of the upper cranium are multi-layered, containing the inner and outer tables of denser cortical bone with a high wave speed, surrounding the diploë, which is formed of cancellous bone with lower density and wave speed. This structure, together with the large contrast in properties between the skull and its surrounding soft tissues, provides the principal mechanism for transcranial signal attenuation, with anelastic absorption and elastic mode-conversions playing a less-significant role^24–26^; in the Supplementary Information, we examine explicitly the significance of absorption and demonstrate its limited impact. The model of the skull used in this study included all cavities, foramina, and other structural complications that are present in the adult human head, and that are capable of being captured on the 500 μm grid that we used to represent the model.

The model recovered by FWI, Fig. 3g–i, is in good agreement with the true model, Fig. 3a–c, for both extracranial and intracranial soft tissues. Inside the skull, FWI is able to generate an accurate and detailed image: grey and white matter match the target tissue properties accurately, both in absolute wave speed and in structure, with sufficient resolution to allow direct identification of cortical folds. Deeper structures such as the corpus callosum, the thalamus, the basal ganglia, and the ventricular system are recovered well. Parts of the venous sinuses have a thickness of 0.8 mm in the true model, as do larger vessels within the brain, and these are recovered in the reconstructed image, demonstrating that we are able to achieve sub-millimetre resolution of the brain and its vascular system using only relatively low frequencies lying below 1 MHz. Parts of the cerebellum and the pons lie inferior to the lowest transducer positions in our numerical experiment, but it is still possible to extract sufficient information from the data to image both bodies, although there is a decrease in resolution as illumination is progressively lost in the area close to the base of the skull.

### Recovering the brain without an a priori model of the skull

FWI is a local optimisation algorithm that requires an initial model that lies within the basin of attraction of the global solution^15^. The variation in soft-tissue acoustic wave speed is ~ ± 7%, which has values between ~1400 ms^−1^ for fat and 1600 ms^−1^ for muscle tissue and cartilage^27^. At the frequencies that we use for FWI, such relatively small perturbations are readily retrievable starting from a homogeneous model having a wave speed similar to that of water at ~1500 ms^−1^, as demonstrated in Fig. 2. This is the reason why FWI applied, for example, to breast imaging has been immediately successful^23,28^. In contrast, the variation in wave speed for hard tissue in the cranium is larger at ~ ±14%, with values between ~ 2100 ms^−1^ for cancellous bone and 2800 ms^−1^ for cortical bone^24–26^; the mandible and the vertebrae have even higher wave speeds of ~3500 ms^−1^^27^. These high values are far removed from that of water. Consequently, recovery of the full model of the head, including the bones of the skull, requires a more-sophisticated approach; we show in this section that this can be provided by the AWI algorithm^3^.

**Fig. 4:**
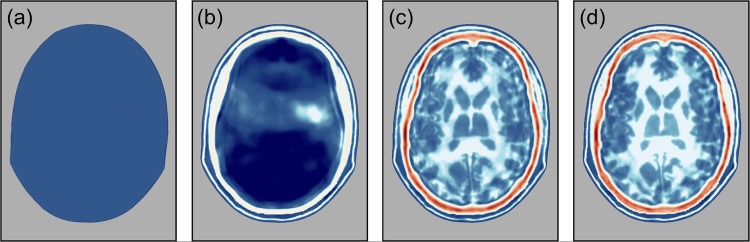
Inverting from a homogeneous starting model. **a** Homogeneous starting model with velocity of water. **b** Model recovered using conventional FWI. **c** Model recovered using AWI. **d** Model recovered by FWI following AWI. The colour scale is as shown in Fig. 3. Note that the skull is not present in the starting model, and the final model is well recovered.

Figure 4b shows the failure of an attempt to recover a model of the head using conventional FWI beginning from the purely homogeneous starting model shown in Fig. 4a. This should be compared with Fig. 3g, which shows the analogous result obtained when the starting model contains an accurate model of the skull. The reason for the failure of this attempt is that conventional FWI will not converge to the correct global solution if inversion begins from a model that is too far removed from the true model. Specifically, for successful FWI, the data generated by the starting model must not be shifted in time by more than half a wave cycle at the lowest frequencies that are present in the data. When that condition is not met, the data are “cycle skipped”, and FWI will then typically fail.

AWI is a modification of FWI that is less sensitive to the quality of the starting model, and it is able to move towards the true model even when the data are cycle skipped. It does, however, pay a price for this robustness, and the models that it is able to produce on its own are not normally as well resolved as those that can be produced by FWI. The solution then is to begin with AWI from a simple homogeneous cycle-skipped model, then switch to conventional FWI once AWI has moved the model sufficiently far towards the true model that they are no longer cycle skipped^19^.

Figure 4c shows the result of applying AWI, using the same data and starting from the same homogeneous model as was used to generate Fig. 4b. Now the attempt to recover a model of both the skull and the brain has been reasonably successful, and no a priori model of the skull has been assumed. The AWI model though is not the final result. Figure 4d shows the results subsequently obtained by conventional FWI beginning from a smoothed version of the model previously recovered by AWI. The final model recovered by this combination of methods is now accurate and compares well to the model in Fig. 3g that was recovered using a perfect model of the skull. AWI and FWI together then can fully solve the problem of building a well-resolved accurate model of skull and brain purely from ultrasound data without any a priori knowledge of the skull.

### Importance of three dimensions

Most three-dimensional medical imaging analyses data initially in two dimensions in order to produce a stack of planes that are combined to form a final 3D image volume. There would be advantages in applying this approach to ultrasound FWI: the computational cost of inverting many 2D slices is lower than that of true 3D inversion, and 2D acquisition systems are simpler to design, build, and operate. However, the structural complexities of the skull, and large contrast with soft tissue, act to distort the wavefronts by refracting and scattering energy out of a 2D plane.

**Fig. 5:**
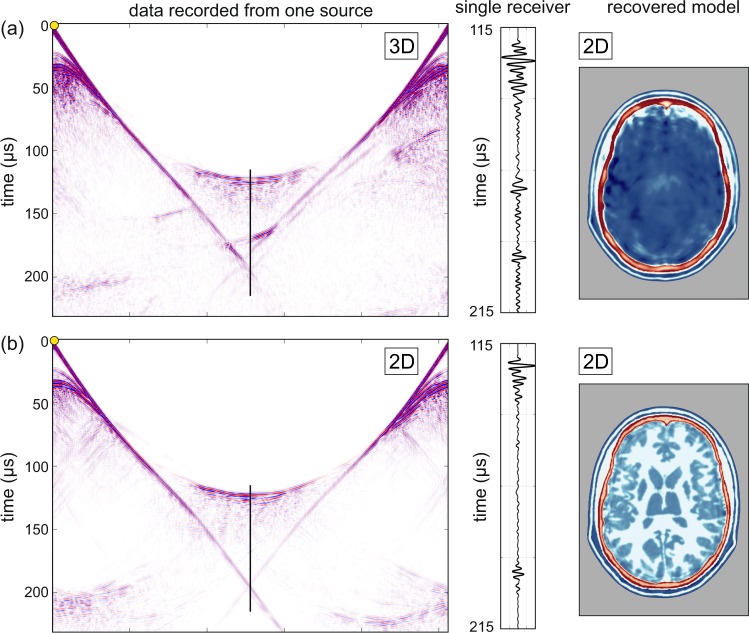
Model recovery using two-dimensional FWI. **a** 3D data inverted using 2D FWI. **b** 2D data inverted using 2D FWI. Left panels show simulated data generated by a single source located at the yellow circle, as recorded on an elliptical array of 512 transducers placed around the head. Centre panels show data recorded by a single receiver located opposite the source; the position of the data shown is indicated by the blue line. Right panels show models recovered using purely two-dimensional FWI. Colour scale as shown in Fig. 3.

Figure 5a illustrates the detrimental effects of inverting three-dimensional data in only two dimensions. Here, the data being inverted are a dense two-dimensional subset of the full three-dimensional data used to generate the results shown in Fig. 3. In Fig. 5a, the data to be inverted have been generated by a 3D wave equation applied to a 3D model, but the inversion assumes only a 2D model and uses a 2D wave equation. The inversion is therefore unable to explain energy that has been refracted, reflected, scattered, or guided out of the 2D plane. The model recovered in this case is neither accurate nor useful.

Figure 5b shows the equivalent experiment conducted purely in 2D; in this second case, both the initial data generation and the inversion are two-dimensional. The 2D inversion of 2D data recovers a model that is as accurate as that recovered by 3D inversion of 3D data. Comparing the data and waveforms in Fig. 5a, b demonstrates why 3D FWI of 3D data, and 2D FWI of 2D data both succeed, whereas 2D FWI of 3D data fails entirely. The 2D and 3D data sets show major differences, and it is evident that there must be significant out-of-plane energy present in the 3D data, and this cannot be explained adequately during 2D FWI. As three-dimensional effects will always be present in real data, successful imaging of the brain using transmission FWI will always require 3D data acquisition and 3D inversion in order to correct properly for the three-dimensional distortion of the wavefield produced by the bones of the skull.

### In vivo laboratory observations

At the sub-MHz frequencies that were used in our numerical experiments, transmission losses in soft tissue are small^27,29–34^, but scattering and anelastic losses in the skull can be important^24–26^. To test the significance of these losses, and to measure signal-to-noise ratios in real transcranial ultrasound, we made in vivo and ex vivo laboratory observations. In both these experiments, the transceiver bandwidth and the source waveform were identical to those used in the in silico simulations. Ultrasound intensities were at all times below the lowest limits recommended by the British Medical Ultrasound Society for continuous adult diagnostic ultrasound^35^. In the in silico and ex vivo experiments, the subject head and the transducers were immersed in water. In contrast, for the in vivo observations, the transducers were held against the scalp, coupled to the subject using sonographic gel. Both in vivo and ex vivo experiments produced similar levels of signal and similar signal-to-noise ratios

**Fig. 6:**
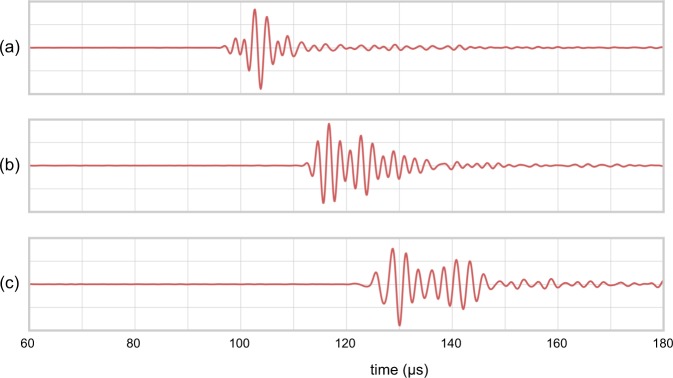
In vivo transcranial observations. Un-processed, un-stacked, transcranial, ultrasound signals, low-pass filtered at 1 MHz, generated using a single source and single receiver, coupled to the subject using sonographic gel, and held firmly against the scalp and hair. The source waveform is identical to that used in both in silico and ex vivo experiments. The sonograms are amplitude normalised; true amplitudes and travel times are approximately proportional to source-receiver separation. **a** Temporal to temporal. **b** Left frontal to right occipital. **c** Frontal to parietal.

Figure 6 shows transcranial in vivo waveforms acquired in the laboratory, at three orientations, using one of the authors as a subject. The data are displayed un-stacked and un-processed; that is, a single unfocused source was triggered once, and the raw data recorded on a single-channel receiver are displayed without beam forming, dynamic compression or other numerical manipulation. These observations demonstrate unequivocally that transcranial ultrasound can be recorded for the in vivo adult human head, with good signal-to-noise ratios, for the 100–850 kHz bandwidth that we have used through this study using the simplest of acquisition systems. These unfocussed signals have travelled across the head, successively through gel, hair, skin, external soft tissue, cranium, brain, and other internal soft tissue, cranium, external tissues, and gel, in a variety of orientations and alignments.

We calculated observed signal-to-noise levels by comparing the RMS amplitude recorded before the transmitted signal arrives with that recorded within a 20-μs window following the first signal arrival. Signal-to-noise ratios calculated in this way for the in vivo data, averaged over all orientations, were 39 dB, with a maximum of 10 dB variation with orientation. We demonstrate in the Supplementary Information that FWI remains robust at signal-to-noise ratios below 2 dB. Our direct in vivo observations thus demonstrate observed signal-to-noise levels that are very much larger than the minimum required for successful FWI, and were obtained using safe levels of incident ultrasound, and employed transducers and electronics that are readily deployable in a clinical setting. It is clear that the signal penetration and signal-to-noise ratios that are likely to be achievable in practical applications at sub-MHz frequencies will be more than sufficient for transcranial neuroimaging using low-frequency ultrasound supported by FWI.

### Ex vivo laboratory observations

**Fig. 7:**
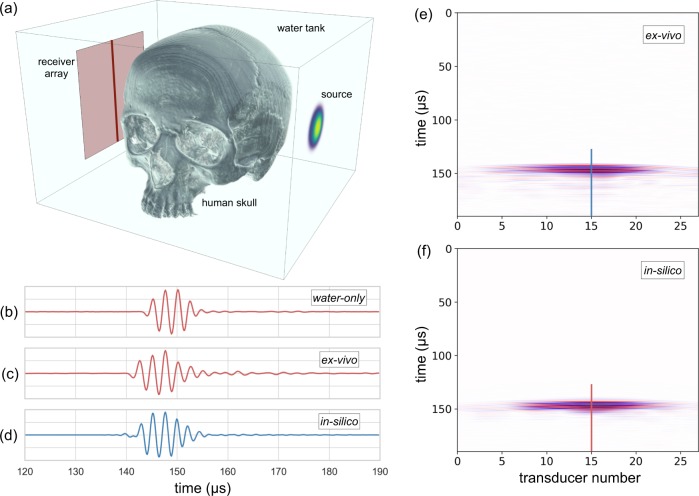
Ex vivo and in silico data after transmission across the head. **a** The geometry of the ex vivo laboratory experiment. **b** Data recorded in the laboratory by the central transducer without the skull present. **c** The equivalent ex vivo data recorded with the skull. **d** The equivalent in silico data with the skull. **e** Laboratory ex vivo data recorded on a finely sampled linear array with the skull. **f** The equivalent numerical data simulated in 3D. The physical skull and the numerical model are nominally the same, but differ in detail, and the numerical model does not include the effects of scattering by the physical transducers and their supporting hardware. Figures **b**–**d** are normalised to their largest amplitude; the un-normalised amplitudes in **b** are about five times larger than those in **c**.

In the ex vivo experiment, we immersed a human skull in water, recording transcranial ultrasound using the same transducers as were used for the in vivo observations. The data recorded, with and without the skull present, are displayed in Fig. 7. The principal signal losses within an in vivo human head occur as the result of anelastic loss within bone, reflection at the inner and outer boundaries of the skull, and scattering within the cranium especially at the boundary between the diploë and cortical bone. The principal wavefront distortions are produced by the large sound speed contrast between the bones of the skull and their surrounding soft tissues, which have a sound speed close to that of water. This ex vivo experiment is designed to capture all those features.

Figure 7b shows the data recorded by a single transducer without the skull present, and Fig. 7c shows data from the same transducer with the skull interposed between the source and receiver. Amplitudes are normalised in these figures so that Fig. 7c is displayed at about five times the gain of Fig. 7b. The noise level in the transcranial ex vivo data set observed in Fig. 7c is low; the signal-to-noise ratio was 34 dB when measured as for the in vivo data. The principal effect of interposing the skull is to advance the arrival time by ~2.5 μs, which corresponds to one cycle at the 400-kHz dominant frequency, and to reduce the transmitted amplitude by about a factor of five. Assuming a skull thickness of 7 mm, physical properties for wave speed, density, and absorption given in Supplementary Table 1, and a cranium in which lower-velocity lower density diploë is sandwiched between faster, denser cortical bone, the observed delay time is as expected. This delay means that the observed data are cycle skipped with respect to a homogeneous model that contains only water, and it explains why simple FWI fails to converge properly when beginning from such a model, as in Fig. 4a.

Using the same simple model of the cranium to calculate anticipated transmission amplitude losses produced by the skull, the principal effect is that caused by normal-incidence reflections at the water-to-cortical bone interfaces and at the cortical-to-diploë interfaces. Together, these eight reflections reduce transmitted amplitudes to ~36% of their values in a homogeneous water model. Amplitude loss, produced by anelastic absorption within both the cortical and cancellous layers, reduces amplitudes at 400 kHz to about a further 77%, so that the total transmitted amplitude would be expected to drop to ~28% as a result of transmission through the skull. In the experimental data, the corresponding amplitude drop is ~22%, well within the uncertainties of this simple model. That the observed losses are slightly greater than the calculated values is most likely a consequence of small-scale structural complexity within the real skull, which will moderately increase the magnitude of internal scattering.

We repeated the same experiment as shown in Fig. 7c, e in silico, using a model obtained by converting a high-intensity X-ray CT image volume of the ex vivo skull into acoustic sound speed. The conversion from X-ray attenuation to sound speed is not exact, so that the in silico data would not be expected to provide an exact match to the laboratory ex vivo data. The real experiment will also contain signals scattered by the physical transducers and their supporting infrastructure; we did not attempt to duplicate these additional signals in silico. Figure 7 compares the two datasets. It shows that the timing, waveform, absolute amplitude and variation of amplitude with position and time, in the ex vivo laboratory data, are well reproduced by the in silico simulation, verifying that our skull model and our modelling assumptions are both reasonable.

### Clinical application to stroke

The application of FWI to neuroimaging has the potential to improve diagnosis in a wide range of neurological pathologies; here, we explore its potential to aid early treatment of stroke, a major cause of death and adult disability worldwide^1^. Stroke has two principal causes: ischaemic stroke is most commonly caused by a blood clot obstructing blood supply to the brain, and haemorrhagic stroke is most commonly caused by bleeding within the brain parenchyma. When the blood supply to the brain is compromised, rapid intervention is required to restore circulatory integrity, halt and reverse tissue damage, and prevent and reduce morbidity, mortality, and disability. Although there are a number of early treatments available, including thrombolysis, mechanical thrombus extraction, and similar interventions^36,37^, their applicability is limited in practice by the requirement for accurate high-resolution brain imaging before these treatments can be deployed^2^.

The indicated treatment for ischaemic stroke is contraindicated for haemorrhagic stroke; brain imaging is therefore required to diagnose and separate these causes. The need for speed is paramount, but MRI is not portable and X-ray CT is barely so. Brain imaging then takes place not when paramedics first reach the patient, not within the ambulance, and not often within accident and emergency units; as a result, relatively few stroke patients receive a brain scan of any kind within the critical first hour, and even fewer receive high-quality MRI^2^. There is then a clear need for portable, fast, high-resolution, high-fidelity, 3D, brain imaging that can differentiate between ischaemic and haemorrhagic stroke, and differentiate these from other pathologies that can mimic stroke. The development and clinical application of such a method would markedly increase the survival rate and reduce the severity of subsequent disability by enabling much earlier treatment at the point of first patient contact.

**Fig. 8:**
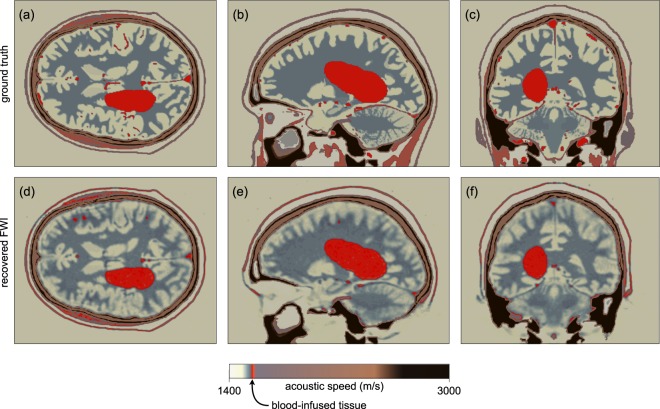
Recovery of a large haemorrhage by FWI. **a**–**c** Slices through a 3D wave-speed model perturbed by a large haemorrhage. **d**–**f** The same slices through the FWI. The haemorrhage is well recovered by full-waveform inversion at high resolution in all slices. The colour scale has been modified to highlight the haemorrhage.

**Fig. 9:**
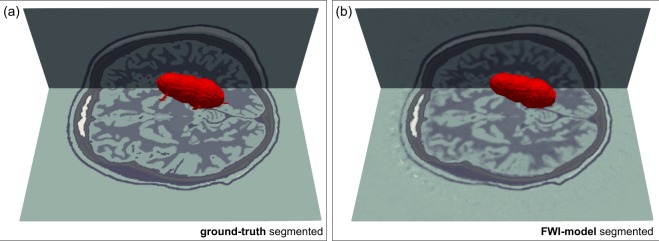
Segmented haemorrhage. **a** Haemorrhage auto-segmented from the true model. **b** Haemorrhage auto-segmented from the model recovered by full-waveform inversion.

To test the viability of this concept, we modified the target model to contain a haemorrhage, Fig. 8a–c. To build this model, we used physical properties for blood-infused soft tissue^38,39^. Using a homogeneous starting model for the brain, and the true model for the skull, we recovered the FWI images in Fig. 8d–f. Figure 9 shows that the haemorrhage can be readily segmented from the three-dimensional model, both in the target and the FWI-recovered models. The target pathology is well recovered in the FWI image, and has good tissue contrast with other features of the brain. The boundaries and exact extent of the pathology are clear; and although it is not shown here, FWI is well able to produce time-lapsed images over a wide variety of time scales from seconds to hours. The spatial resolution of FWI is capable of detecting haemorrhage at all scales down to that of the originating vasculature.

## Discussion

Both X-ray CT and MRI revolutionised medical imaging when they first appeared; three-dimensional transmission and reflection ultrasound tomography using FWI has the same potential for impact across multiple disciplines, and has especial relevance for rapid diagnosis and treatment of stroke. Supplementary Fig. 1 shows that the method is robust against the levels of noise that we observe in realistic ex vivo and in vivo laboratory experiments, and transcranial ultrasound signals have large amplitudes at the relatively low, sub-MHz, frequencies that are sufficient for successful sub-millimetre resolution. The method overcomes the well-known limitations of conventional pulse-echo ultrasound imaging of the adult human brain, and the related limitations of conventional time-of-flight tomography.

It is necessary either to include some prior model of the skull within the starting model before attempting to recover the brain, or to use an advanced form of FWI such as AWI that can converge toward the correct answer from a simple starting model. To account correctly for and remove the distorting effects of the skull, it is essential to acquire and invert the data in three dimensions, and unlike many other imaging techniques, it is not possible to reduce three-dimensional imaging merely to the sum total of a sequence of two-dimensional slices. In Supplementary Fig. 2, we show that, at the low frequencies required by FWI, it is not necessary to include density or anelastic absorption explicitly in the inversion in order to recover a good image, but it may be desirable to do so, both to improve the accuracy of the final image, and to obtain additional independent parameters to aid diagnosis. Other ultrasound methodologies, employing higher frequencies, are more severely impacted by signal attenuation, both in the skull and in soft tissue.

The computational effort required for 3D FWI is considerable. The results shown in Fig. 3 required ~32 hours elapsed time to complete, running on a conventional cluster of 128, CPU-based, 24-core, compute nodes. Our target is to reduce this elapsed time to below 10 minutes; this requires a speed up of ~200 times. The hardware that we used has a peak performance of ~60 tera-flop, so achieving the desired speed up requires hardware capable of operating usefully at a peak of ~12 peta-flop. Individual high-performance GPU-based servers are currently able to achieve speeds in excess of one peta-flop, so that a small array of these would in principle be capable of producing a final model in < 10 minutes. Assuming 2019 prices, amortisation over 3 years, and full utilisation of the hardware, the capital cost of the GPU-server hardware required to do this represents a few tens of dollars to invert a 3D transcranial dataset on a 500-μm grid. So, although the computational burden of FWI is high, the cost per patient to achieve a 10-minute turnaround is not high under appropriate circumstances.

The potential value of FWI imaging is threefold. Most importantly, it could improve outcomes in acute neurological disorders such as stroke and head trauma by enabling earlier intervention; the ultimate aim is diagnosis and treatment within minutes of first contact with paramedics. Second, the low cost, high safety, portability, and high resolving power of the technology provides the ability to monitor the brains of patients continuously at the bedside allowing clinicians to intervene, for a range of pathologies, to prevent injury with the speed that the brain demands, acting in rapid response as if the brain image was a simple physiological variable such as blood pressure. And third, the technology can be deployed readily and safely, for prevention and diagnosis, in a wide range of situations where neuroimaging would be desirable but is currently unavailable—for example, within developing nations with limited health budgets, in remote locations, routinely at contact-sports events, within military deployments, or as part of disaster relief when local infrastructure is compromised.

## Methods

### In silico model

We used the MIDA 3D numerical model of the human head^40^, at the original sample spacing of 500 μm, as the basis to build the sound speed model used in the in silico simulations. Physical properties within the model were derived from the geometry of the segmented model combined with values for acoustic sound speed, density, and absorption for different tissue types from^24–27,29–34^; further details appear in Supplementary Table 1. Most minor tissue types within the model have unmeasured acoustic properties; in these cases, we estimated their values using small perturbations to the properties of other tissue types that appeared analogous in their other physical properties and composition.

The models used to generate the data for all figures except Supplementary Fig. 2 were purely acoustic. The model used for Supplementary Fig. 2 included anelastic absorption, and assumed a linear relationship between attenuation and signal frequency. At the relatively low frequencies used in these simulations, such a model of absorption provides a reasonable approximation to the properties of real tissue^29^.

### In silico modelling

The experimental geometry was restricted to accommodate the application of this technology realistically to human patients in a clinical setting, and therefore no transducers were positioned in front of the face or below the base of the head. Transducers were modelled assuming unfocused single elements. We used 1024 transducers that acted as both sources and receivers, generating just over a million source-receiver records of acoustic pressure, each record lasting 240 μs. The source waveform consisted of a three-cycle tone burst having a peak amplitude at a frequency of 400 kHz and a useful bandwidth for FWI extending from ~100–850 kHz. This source waveform is identical to that generated in our laboratory experiments.

The synthetic data were generated by solving the three-dimensional, variable density, isotropic, linear, acoustic wave equation, explicitly in the time domain, using a time-stepping finite-difference algorithm, with an optimised stencil that is nominally tenth-order in space and fourth-order in time. For the two-dimensional data shown in Fig. 4b, the analogous two-dimensional wave equation was solved in which the model, wavefield, sources, and receivers do not vary perpendicular to a two-dimensional plane. For the anelastic data used to generate Supplementary Fig. 2, the visco-acoustic wave equation was solved using the method described in ref. ^41^.

### Laboratory experiments

The ex vivo laboratory experiment was performed by immersing a formalin-preserved human skull in water, generating an ultrasound pulse on one side of the head, and recording the resultant signals on the other. The skull retained some residual soft tissue, was stored dry, and was typically immersed for a few tens of minutes during ultrasound measurements. In the ex vivo experiment, sources, and receivers were not in direct contact with the skull; in the in vivo experiment, sources, and receivers were in direct contact with the head, coupled using sonographic gel.

For the in vivo observations, we used single-element flat unfocused Blatek-Industries 400-kHz AT30871 10-mm piezoelectric transducers as source and receiver. For the ex vivo experiments, we used a single-element unfocused Olympus 500 kHz V381 19-mm ultrasound source transducer. In both in vivo and ex vivo experiments, the transducers were driven to generate a three-cycle tone burst centred on 400 kHz at the intensities commonly employed in conventional medical imaging^7^; this waveform matched that used in the in silico experiments. For the ex vivo experiment, we recorded the transmitted signals from the source using an Olympus 500 kHz V301 25-mm transducer located on the opposite side of the skull at a perpendicular distance of 210 mm from the source. A planar receiver array was formed by moving the single receiving transducer successively in 4-mm steps to 729 positions to form a 27 × 27 array measuring 108 × 108 mm. For both the in vivo and ex vivo experiments, the data were generated and recorded using a Verasonics Vantage 256, and the recorded data were low-pass filtered at 1 MHz.

The in vivo experiment employed one of the authors as a participant, for whom informed consent was obtained prior to the experiment. The experiments complied with all relevant ethical regulations; the study was approved by the Joint Research Compliance Office and the Imperial College Research Ethics Committee, both of Imperial College London.

### FWI iteration

During FWI, we inverted the in silico data in the time domain over eight finite-frequency bands, starting at a dominant frequency of ~ 100 kHz, and moving successively to higher frequencies to reach the maximum useable frequency in the data of ~850 kHz. This multi-scale approach helps to ensure that the inversion does not become trapped at some local solution produced by inadequacies in the starting model. Within each frequency band, we used ten iterations in total, using a tenth of the data at each iteration, so that data from each source was used just once per frequency band. The highest frequencies present in the data have a half-wavelength in the brain of less than a millimetre, so that we would expect to be able to resolve sub-millimetre structure in the final recovered model.

During AWI, we began the inversion from a homogeneous water model using a single frequency band centred on 100 kHz. Other than the change of misfit function, AWI proceeds in a similar fashion to FWI. Following AWI, we smoothed the resultant model, and used that to begin FWI from 320 kHz, moving successively to the maximum frequency in the data as before.

### FWI algorithm

FWI is an imaging method that seeks to find a model that can numerically reproduce experimental data. It does this by solving a non-linear least-squares local optimisation problem, modifying the model in order to minimise the misfit, *f*, defined as (half) the sum of the squares of the differences between the experimental data and an equivalent simulated dataset that is numerically generated using a numerical model of acoustic properties. Thus, we seek to minimise:

1 $$f = \frac{1}{2}\left( {{\mathbf{p}} - {\mathbf{d}}} \right)^{\mathrm{T}}\left( {{\mathbf{p}} - {\mathbf{d}}} \right)$$

where **p** and **d** represent the predicted (numerically generated) and observed (experimental) data, respectively, organised as vectors that contain concatenated time-series of pressure variations at each recording location for every source location. In this in silico study, the experimental data were themselves generated using a known ground-truth target model. At the initiation of FWI, a starting model **m**, representing an initial estimate of the target of interest and composed of many model parameters *m*_*i*_, is used to solve the wave equation and generate the predicted data set **p**. In the account below, the quantities *f*, **p**, **A**, and **u** each depend upon the assumed model **m**, whereas **d**, **s**, and **R** do not.

The computational cost of numerically solving the governing wave equation in 3D for many sources restricts computationally tractable solutions to iterated local gradient-descent methods. We solve the problem by seeking the direction of steepest descent, on the hyper-surface defined by *f*, which has as many dimensions as there are model parameters *m*_*i*_. For the 3D model shown in Fig. 3, this hyper-surface had ~ 10^8^ dimensions. This gradient-descent algorithm seeks to move from the starting model, by a sequence of small steps, successively downhill on this hyper-surface, to arrive close to the model that lies at the lowest point on the surface—this is the model that best predicts the observed data in a least-squares sense.

In order to find the direction of steepest descent, the derivative of the misfit with respect to the model parameters is found using the adjoint-state method^15^. The derivative of *f* with respect to each of the model parameters *m*_*i*_ takes the form:

2 $$\frac{{\partial f}}{{\partial m_i}}\;=\;\left[ {\frac{{\partial {\mathbf{p}}}}{{\partial m_i}}} \right]^{\mathrm{T}}\left( {{\mathbf{p}} - {\mathbf{d}}} \right)$$

To compute the first derivative of the predicted data with respect to the model parameters *m*_*i*_, we start by writing the wave equation as a matrix-vector operation:

3 $${\mathbf{Au}} = {\mathbf{s}}$$

where **A** is the wave equation written in a suitable discrete form—here we used high-order finite differences to approximate the 3D anisotropic variable density visco-acoustic wave equation, **u** is the pressure wavefield and **s** is the source. Differentiating this with respect to *m*_*i*_, and taking into account that both **A** and **u** depend upon the model parameter *m*_*i*_ but that the source **s** does not, gives:

4 $${\mathbf{A}}\frac{{\partial {\mathbf{u}}}}{{\partial m_i}}\;+\; \frac{{\partial {\mathbf{A}}}}{{\partial m_i}}{\mathbf{u}}={\mathbf{0}}$$

Assuming that **A** is invertible, which it must be if Eq. 3 has a unique solution, leads to the expression:

5 $$\frac{{\partial {\mathbf{u}}}}{{\partial m_i}}\;=\;- {\mathbf{A}}^{ - 1}\frac{{\partial {\mathbf{A}}}}{{\partial m_i}}{\mathbf{u}}$$

for the variation of the wavefield **u** with the model parameter *m*_*i*_. Now, the predicted data **p** are simply a subset of the full wavefield obtained at those locations where we happen to have placed receivers. Thus, we can use a restriction matrix **R** to extract the corresponding data as **p** = **Ru**, so that:

6 $$\frac{{\partial {\mathbf{p}}}}{{\partial m_i}} = - {\mathbf{RA}}^{ - 1}\frac{{\partial {\mathbf{A}}}}{{\partial m_i}}{\mathbf{u}}$$

Because, again, **R** does not depend on *m*_*i*_. The final expression for the gradient is then:

7 $$\frac{{\partial f}}{{\partial m_i}}\;=\;- {\mathbf{u}}^{\mathrm{T}}\frac{{\partial {\mathbf{A}}^{\mathrm{T}}}}{{\partial m_i}}{\mathbf{A}}^{ - {\mathrm{T}}}{\mathbf{R}}^{\mathrm{T}}\left( {{\mathbf{p}} - {\mathbf{d}}} \right)$$

Reading from right to left, this expression implies the following sequence of steps to calculate the gradient:

1. Compute the residual data (**p** – **d)** by solving the wave equation to generate **p** in the starting model,
2. Inject the residual data into the model at the receiver positions,
3. Solve the wave equation backwards in time using the injected residual data as a virtual source,
4. Scale the resulting wavefield using the differential of the wave equation operator **A** with respect to the model parameters *m*_*i*_,
5. Find the zero lag of the cross-correlation of this wavefield in time with the original forward wavefield **u** at every point in the model.

The result of applying these steps is a gradient vector oriented to point in the direction of maximum increase of *f* at the current position in the solution space. The negative of the gradient indicates the direction in which small changes to the model will create the largest decrease in the misfit *f*, so the model should be changed in this direction. Typically, the problem will be non-linear and non-convex, and therefore it requires that the model is updated iteratively, and that the starting point is within the basin of attraction of the global minimum. In a practical FWI algorithm, the direction of steepest descent is typically preconditioned in some way to speed convergence; here, we used spatial preconditioning to compensate for illumination variation within the model^16^. A more-complete development, and further details are given in ref. ^15,16,18^.

### AWI algorithm

AWI^3^ is a form of FWI that has immunity to cycle skipping. In conventional FWI, the algorithm seeks to drive the sample-by-sample difference between the predicted and observed data to zero. In contrast, in AWI the algorithm seeks to drive the ratio between the two data sets to unity. Both approaches aim to drive the predicted data set towards the observed data set, and consequently to drive the recovered model towards the true model. With perfect data, and perfect algorithms, both approaches will reach the same end point. However, when both methods are implemented using local gradient descent, they follow different paths through the space of possible models in their attempt to reach the true model. In these circumstances, FWI will tend to become trapped in local minima when the predicted data differ from the observed data by more than half a wave cycle, whereas AWI will not.

The ratio used in AWI is not generated sample-by-sample; rather it is a ratio formed frequency-by-frequency after temporal Fourier transform, and the ratio is formed separately for each source-receiver pair. As division in the frequency domain represents deconvolution in the time domain, the AWI algorithm effectively deconvolves one data set by the other and then attempts to drive the result of that deconvolution toward a unit-amplitude delta function at zero temporal lag. The mathematical details are given in ref. ^3^, and practical details are given in ref. ^19^.

### Algorithm for time-of-flight tomography

The model shown in Fig. 2c was generated using time-of-flight tomography. This method is analogous to FWI, but with two significant differences: the data to be inverted consist of single numbers, one for each source-receiver pair, representing the time taken for energy to travel through the target model from source to receiver; and geometric ray-tracing rather than the full wave equation is used to calculate these travel times. This approach has the advantage that it is computationally more tractable than FWI, and so runs orders of magnitude more quickly; it is also much less likely than FWI to become trapped in local minima. But, it has the disadvantage that it cannot recover structure in the model below the scale of the diameter of the first Fresnel zone.

We solved the tomographic problem by minimising a misfit similar to that shown in Eq. (1), but where **p** and **d** are now vectors containing the travel times rather than the raw observed acoustic pressure data. We address the problem as before by solving a non-linear least-squares local optimisation problem, using gradient descent preconditioned by conjugate gradients. Unlike the more familiar X-ray CT, in ultrasound tomography changes to the model affect the path that energy follows from source to receiver, and it is necessary to include this non-linear effect into the inversion by iterating.

### Reporting summary

Further information on experimental design is available in the Nature Research Reporting Summary linked to this article.

## Supplementary information

Supplementary Information

Supplementary Movie 1

Supplementary Movie 2

Supplementary Movie 3

Supplementary Movie 4

Reporting Summary

## Data Availability

The true model was generated using the MIDA model described in ref. ^40^. The model can be obtained in digital form from the authors of that paper. We populated that model using the physical properties shown in Supplementary Table 1. The synthetic acoustic dataset that we generated from this model is available from the Dryad digital archive (10.5061/dryad.nzs7h44n7).

## Code availability

The computer code used to generate synthetic data and to perform the inversions is Fullwave3D, described in ref. ^16^. This is a large and sophisticated commercial package developed for and licensed to the petroleum industry. The software is available on commercial terms, and can be licensed both for use on private HPC systems and on the commercial public cloud. For those wishing simply to reproduce the results shown here, a more-practical option is likely to be to use one of the several open-source modelling and FWI packages that are now becoming available, or for those with access to commercial geophysical processing software to use one of the growing number of such packages that include facilities for performing FWI. Several of the latter packages are available to university-based researchers, for non-commercial use, on heavily discounted terms.
