# Supplementary material for: Full-waveform inversion imaging of the human brain
Source: NPJ Digit Med. 2020 Mar 6;3:28. doi: 10.1038/s41746-020-0240-8 (PMC7060331; doi:10.1038/s41746-020-0240-8)
Supplement: Supplementary file 1 — Supplementary Information [file 41746_2020_240_MOESM1_ESM.pdf]

SUPPLEMENTARY INFORMATION

Full-waveform inversion imaging of the human brain

Authors: Lluís Guasch[1*], Oscar Calderón Agudo[1], Meng-Xing Tang[2], Parashkev Nachev[3], Michael Warner[1]

[*] *Corresponding author*

[1] Department of Earth Science and Engineering, Imperial College London, London, SW7 2AZ, UK.

[2] Department of Bioengineering, Imperial College London, London, SW7 2AZ, UK.

[3] Institute of Neurology, University College London, 33 Queen Square, London, WC1N 3BG, UK.

Contact: l.guasch08@imperial.ac.uk

SUPPLEMENTARY VIDEOS

Supplementary Video 1. **Synthesized wavefield crossing the head.** The wavefield from Fig. 1, generated by a single emitter, is shown in time as it propagates across the head.

Supplementary Video 2. Transverse planes. The target, starting and recovered models from Fig. 3 are shown in three dimensions using a sequence of transverse slices.

Supplementary Video 3. Sagittal planes. The target, starting and recovered models from Fig. 3 are shown in three dimensions using a sequence of sagittal slices.

Supplementary Video 4. Coronal planes. The target, starting and recovered models from Fig. 3 are shown in three dimensions using a sequence of coronal slices.

SUPPLEMENTARY RESULTS

Effect of noise on FWI

Our *in-silico* conclusions are only relevant to the real world if ultrasound signals, of the intensity required for medical imaging and with the bandwidth that we use here, can be recorded with sufficient signal-to-noise ratio after propagation across the head, traversing the bones of the skull twice in the process. Two questions are relevant: how sensitive is FWI to noise, and what level of signal-to-noise can be expected for transcranial ultrasound at the frequencies required? Below, we address the first of these questions; Figs. 6 & 7, in the main text, answered the second question – observed transcranial signal-to-noise ratios in the laboratory are around 35 dB.

Supplementary Fig. 1 shows the results of adding random noise to the *in-silico* data before subsequent inversion. Signal-to-noise noise levels in the transcranial portion of these data are about 1.8 dB or worse, when measured on the same basis as was used to describe the in-vivo and *ex-vivo* laboratory data in Figs. 6 & 7. Apart from the addition of noise, Supplementary Fig. 1 is exactly analogous to Fig. 5b. Comparison of these two figures demonstrates that models recovered with and without the addition of noise are similar; even the high level of noise applied here causes only modest degradation of the reconstructed image.

FWI using transcranial ultrasound is clearly robust in the presence of high levels of incoherent noise. This robustness occurs because the formalism of FWI captures only those parts of the observed data that are capable of being reproduced by application of the wave equation to a model. Most forms of noise cannot be substantially reproduced in this way, and so, while noise may slow down convergence to the correct model, the level of noise in the final model is typically much lower than the level of noise in the data.

Effect of absorption, density, anisotropy and elasticity

The modelling and inversion shown in Fig. 3 in the main text was performed without regard to anelastic absorption, density variation, anisotropy or elastic effects. Supplementary Fig. 2 shows the effects of ignoring both density and absorption during FWI. Absorption and density models for the head are shown in Supplementary Figs. 2a and 2b respectively. Here, absorption is displayed using quality factor Q, defined as 2π times the fraction of energy lost per wave cycle; lower Q values represent higher values of attenuation. Our model here assumes a linear relationship between attenuation and frequency, but it is straightforward to incorporate a more complicated relationship where appropriate.

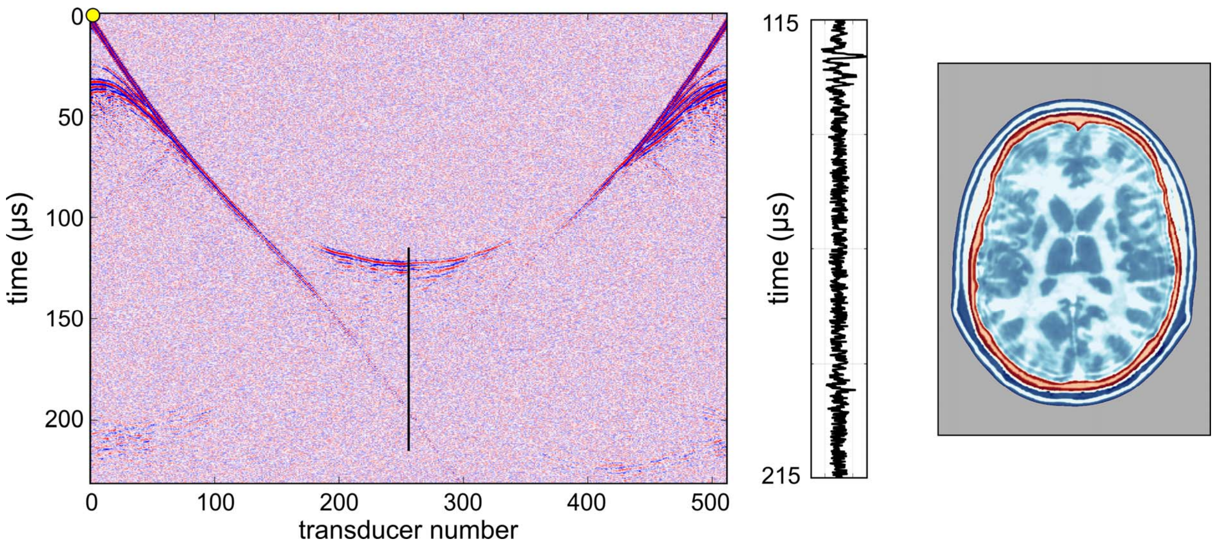

Supplementary Figure 1. Model recovery using imperfect data. Fig. 5b presented noise-free 2D data inverted using 2D FWI. Here, we show the same data and the results of the same inversion, but after the addition of random noise to the raw data. The noise level in this test is much higher than the level of noise observed in the *in-vivo* and *ex-vivo* experiments, and the recovery of the model shows only minor degradation as a consequence of the noise.

Supplementary Fig. 2c shows the model recovered by FWI assuming constant density and no attenuation when inverting simulated data that include both effects. A reasonable representation of the true wave-speed model is recovered. The principal effect of ignoring density variation is to ascribe all variation in signal amplitude to velocity variation, and this tends to exaggerate differences between locally heterogeneous regions. The principal effect of ignoring absorption and its associated velocity dispersion is to reduce the mean sound speed of the recovered model. Consequently, Supplementary Fig. 2c is slower on average than the true model, but its structure is nonetheless accurate. Ignoring both density and absorption effects during inversion is not significantly worse that ignoring either one alone. In clinical application, both of these effects can be included within the inversion – this is straightforward to achieve, but appears not to be necessary for the recovery of a simple well-resolved image. It is also possible to use FWI to invert explicitly for attenuation and other parameters [15], and some pathologies may be more readily diagnosed using such multi-parameter inversion than by use of acoustic wave speed alone.

Anisotropy in wave speed is routinely incorporated into FWI in geophysics [16] because it needs to consider crystalline, micro-fractured and finely layered materials. Other than perhaps in the skull, anisotropy in sound speed is unlikely to significantly affect ultrasound neuroimaging. Fully-anisotropic inversion for the skull is straightforward to incorporate into medical FWI, and most established 3D FWI codes will already have that capability incorporated.

Elastic mode-conversions in soft tissue are small; the skull however does have a significant shear modulus [24, 25], and so can produce elastic effects in acoustic data. We have not yet investigated the quantitative importance of these phenomena in neuroimaging, but we do not expect them to be more significant than the effects shown in Supplementary Fig. 2c. Ultrasound sources and receivers in water do not generate or record shear waves, and elastic conversions are small close to normal incidence, which is the portion of the wavefield that is most important for FWI, both in transmission and reflection imaging. In geophysics, despite

its potential importance, commercial FWI is seldom performed using the full-elastic wave equation, and the resultant models match well to in-situ direct measurements made within boreholes [15-17]. The Fullwave3D code used in this study is able to undertake anisotropic full-elastic 3D FWI, but its significant increased computational cost has not-often proven to be justified, at least in commercial geophysics.

In summary, acoustic, isotropic, constant-density, non-absorbing, three-dimensional inversion appears to be adequate for the generation of well-resolved detailed images of the brain. However, a more-complete account of the full physics during FWI, including absorption and anisotropy in bone, and elasticity in hard tissues, may help to provide a more quantitatively accurate model of physical properties, but at an increased computational cost which, at least initially, will translate into an increase in the total elapsed time required to compute the final model. Practical solutions are likely to involve simple fast acoustic inversion to form an initial image, followed optionally by more-accurate inversion using more-complete physics subsequently as circumstances require. In medical imaging, it is possible that additional physical properties recoverable by FWI will have diagnostic value advantages in special circumstances, as they do in both medical attenuation tomography [42] and in geophysics [43].

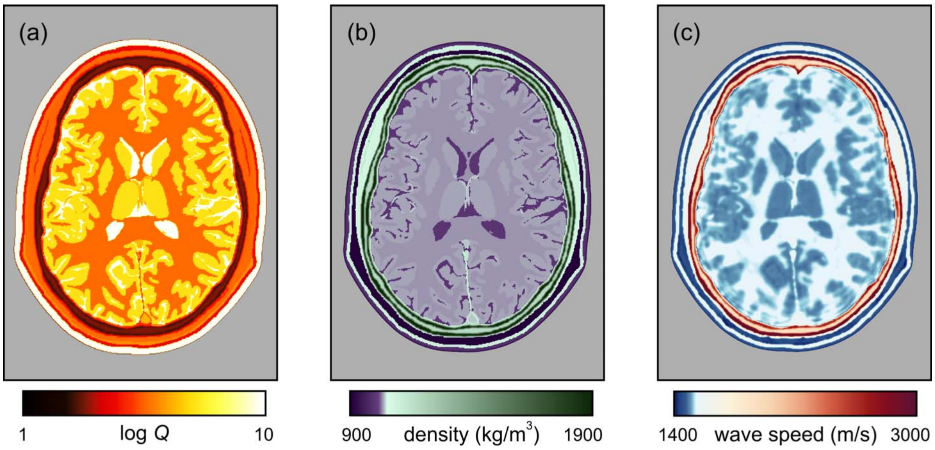

Supplementary Figure 2. Models of the head. (**a**) Model of anelastic absorption. (**b**) Density model. (**c**) Sound speed recovered by FWI, using data generated including absorption and density, but assuming constant density and no attenuation during the inversion. Simulations and inversions are both in 2D.

SUPPLEMENTARY DISCUSSION

Spatial resolution in ultrasound observations

Spatial resolution in conventional pulse-echo ultrasound sonography in the axial direction depends upon pulse duration, and in a lateral direction upon the width of the insonifying beam [20]. In the axial direction, spatial resolution refers to the minimal distance that can be distinguished between two ultrasound reflectors that are oriented perpendicular to the direction of propagation of the ultrasound beam, or equivalently that lie parallel to the scanning direction. This distance is equal to about half the spatial pulse length. The ultrasound pulse used to generate the results shown in Figs. 2 & 3 had three cycles and a dominant frequency of 400 Hz, so that the axial resolution of the corresponding pulse-echo

image would have been about 5.6 mm. The highest frequency in the signal was about 850 Hz, and it is possible to damp real transducers to generate a pulse as short as two cycles. In those circumstances, and assuming a pulse in which this higher frequency dominated, the axial resolution would have been about 1.8 mm.

In directions that are lateral to the beam, spatial resolution in scanned pulse-echo images refers to the minimal distance that can be distinguished between two reflective features that are displaced perpendicular to the direction of propagation of the ultrasound beam, or equivalently that are displaced parallel to the scanning direction. This distance is equal to about that of the width of the insonifying beam at the target distance. Typical transducer systems used for pulse-echo imaging use focused beams. In such systems, the beam width first narrows with distance from the transducer, and then subsequently broadens with increasing distance as the beam diverges beyond the focal length of the system. The width of the focused beam in such a system is approximately equal to its focal length times the wavelength of the signal divided by the aperture of the focusing transducer array.

Throughout this paper, in the numerical experiment used to generate the results in Figs. 2 & 3 specifically, and in FWI generally, focused beams are not employed. However, had such a system been used, with an aperture of about 50 mm (the largest that might realistically have been used to apply pulse-echo imaging to the brain), using a focal length of about 100 mm so that it could image deep into the brain, then its lateral resolution would have been about 7.5 mm at 400 kHz and about 3.5 mm at 850 kHz.

Resolution in pulse-echo images refers to the spatial discrimination of features seen in a reflection image around the location from which the ultrasound energy is reflected. In contrast, in transmission tomography, whether inverting the delay times used by conventional time-of-flight tomography, or the waveforms used by wave-equation FWI, it is the spatial discrimination of features by their effect on the transmission properties of the wave through the medium that is relevant. For time-of-flight tomography, in order to separate two adjacent features within a model, their effect on the recorded delay times must be distinct from the effect of a single feature that represents a smooth mixture between the two adjacent features. And for FWI, the separation of two features requires that their effect on the recorded waveforms must be distinct.

For transmitted energy, a Fresnel zone represents that region of a model through which energy can travel from a source to a receiver, arriving within the same half cycle. Such energy cannot be separated in terms of its delay time, and all such energy contributes to the same time of flight. The first Fresnel zone corresponds to the region of the model through which energy travels that arrives within half a cycle the geometric ray arrival, which corresponds to the arrival time of an infinite-frequency wave travelling along an infinitesimally thin ray path. For a homogeneous model, the diameter of the first Fresnel zone at the midpoint along a path of length x is $\sqrt{x\lambda}$, where λ is the wavelength of the signal. Since details below the scale of the first Fresnel zone do not change observed delay times, the Fresnel-zone diameter provides a limit to the spatial resolution that is obtainable by any method that uses only times of flight to determine the model [11, 22].

FWI however is not limited in this way. It seeks to match waveforms, and these are sensitive to sub-Fresnel-zone structure. In this case, it is the wavelength, not the Fresnel zone that is significant [19, 21]. Energy scattered from two objects that are separated by more than half a wavelength are distinguishable in terms of their waveforms, in at least some directions, from energy scattered from a single composite object. Provided only that the angular coverage of the target region is sufficient, the spatial resolution of transmission tomographic FWI is of the order of half the incident wavelength in all directions. In practice, sufficient angular

coverage can be obtained either by locating sources and receivers at all azimuths, or by making use of multiple scattering in a highly heterogeneous medium. In the present context, FWI takes advantage of both approaches.

For neuro imaging as discussed here, the characteristic sound speed is about 1500 m/s, and the maximum frequency employed is about 850 kHz, having a wavelength of about 1.8 mm in the brain. The separation of source and receiver required for transcranial tomography is about 200 mm, giving a Fresnel-zone diameter at the centre of the head of about 19 mm. This is the resolution to be expected from conventional time-delay ultrasound computer tomography at 850 kHz, and is the reason that such tomography is not useful for brain imaging. The corresponding resolution for FWI is half the wavelength, that is about 0.9 mm. This is about 20 times better resolved in linear dimension, and about 8,000 times better resolved volumetrically. It is the reason that FWI succeeds to image the brain while time-of-flight tomography cannot.

For conventional time-of-flight tomography to reach the resolution of FWI, time-of-flight tomography would need to use signals that have a Fresnel-zone diameter of about 1 mm. Such signals would require frequencies of several hundred MHz, and these lie far above the bandwidth of signals than can cross the head.

For conventional pulse-echo imaging to reach the resolution of FWI in the axial direction would require a central frequency of about 1.7 MHz using a two-cycle tone burst and about 2.5 MHz using a three-cycle burst. To reach the resolution of FWI in a lateral direction in a scanned pulse-echo image using a 50-mm aperture transducer array would require a central frequency of about 3.5 MHz, or more than eight times higher than the central frequency required by FWI.

Practical deployment

Transducer deployment and design

Figure 1a shows the arrangement of sources and receivers used for the in-silico simulations. Physical ultrasound devices employing broadly this geometry are already in clinical use where they are used at high intensities for therapy rather than for imaging. There are two principal designs:

- In the most-commonly employed design [44], a helmet containing the transducers surrounds the head, and the transducers do not directly contact the scalp. The helmet is filled with ionised bubble-free water, and a neoprene seal is used around the face and the neck so that there is no air within the system. This is essentially the design that we have simulated *in-silico*, and is perhaps the design most likely to be employed within hospitals.
- In an alternative design [45], the helmet contains axially movable transducers that are pushed firmly against the scalp. In such a system, air is excluded between the transducers and the head by using sonographic gels that are widely used in conventional ultrasound imaging. We suspect that this will prove to be the more-robust and portable design for imaging outside a hospital setting.

Other possibilities for clinical deployment can be conceived, but in all cases air would be excluded between the sensors and the body. Our approach to FWI brain imaging is agnostic as to which of these systems is used, and in all probability both types will be employed as circumstance demand.

Unlike conventional pulse-echo imaging, where transducer arrays and other design features are used to generate and focus an ultrasound beam, in our application we use single transducers and no such focusing is employed or required. Ideally such transducers would

act as point sources, but in practice their finite dimensions are significant, and these dimensions have to be correctly simulated during FWI; it is straightforward to do this numerically. In conventional real-time ultrasound imaging, computationally intensive post-acquisition processing is not available to modify the characteristics of the transducers digitally. In contrast, during FWI, source and receiver transducer characteristics can be modified extensively during post-processing and inversion. This means that the physical transducer characteristics are less significant than they would be for pulse-echo sonographic imaging. The key parameters are their temporal bandwidth, their sensitivity, their linearity, and their spatial radiation pattern.

The transducers used in the laboratory, and simulated in the *in-silico* experiments, are conventional cylindrical composite piezo-electric devices, with a nominal resonant frequency of 500 kHz, damped and driven to generate a three-cycle bursts with a dominant frequency of 400 kHz. In the laboratory experiment, we used single-element unfocused Olympus 500 kHz V381 19-mm ultrasound source transducers, and used Olympus 500 kHz V301 25-mm receiver transducers though we could have used similar transducers equally effectively for both source and receiver.

Portability and speed of acquisition

Ultrasound systems are typically lightweight, low-volume and low power. Our current laboratory system, which was not designed with portability in mind, consists of a single multi-channel ultrasound recording and control system, a supporting PC and an array of sensors within a 3D-printed helmet. In total it weighs about 30 kg and consumes about 350 W. It would not be difficult to reduce those numbers significantly. A practical portable would consist of two elements: a helmet similar in size and weight to a typical motorcycle helmet, connected by a single cable to the controlling electronics which would resemble a large laptop; the total weight would likely be less than 10 kg.

The propagation of signals across the head takes less than about 200 μs. Typically these signals will reverberate for a few times that duration, so that successive sources can be safely activated about every 2 ms without compromising signal-to-noise ratios. The system uses 1024 source, each of which is activated once. Acquisition of a complete dataset therefore requires only about two seconds of elapsed time in total. In practice, it is likely to be useful to activate individual sources several times in order to add robustness and to assist transducer registration. It may also be desirable to trigger acquisition between heartbeats. With these additional considerations, data acquisition will still be complete within a few tens of seconds.

With current technology, the principal bottleneck is that related to data transfer from the ultrasound system to the computer system that performs the FWI. The total, uncompressed, volume of data acquired will be about 2 GB, and with sensible data selection and compression, that volume is likely to be a few hundred MB. FWI can begin as soon as about 1% of that volume has been received, and data from all sources can be used when about 10% of it has been received. In a hospital or other well-connected location, moving this volume of data quickly is straightforward. In urban locations that are well served by mobile-phone transducers, these data volumes are unlikely to be problematic especially if these communications can prioritised. The connectivity of the world is ever accelerating, and it seems likely that moving the necessary data volumes for FWI will rapidly become feasible from an ever greater range of locations. In all locations, uplink via satellite is a feasible if costly high-speed option. In all cases, the processing power required for FWI would be accessed remotely, almost certainly in a cloud-based distributed environment.

Speed of FWI computation

FWI computer codes are sophisticated and require several features from their hardware if they are to be sufficiently fast; all those features appear to be present in the most-recent range of GPU-based servers designed for high-performance numerical computation. The devices that can achieve this currently retail at about USD100,000 each. They are 2-PetaFLOP GPU-based servers, with a total of 512 GB of GPU memory distributed across 8 GPUs, containing in total 81,920 cores, 30 TB of SSL disk and 100 Gbps InfiniBand communications.

For 3D brain FWI, propagating and storing the forward wavefield in memory, generating the residual data and back-propagating these residuals through the model, and then cross-correlating and generating the gradient, is readily achievable for one source in less than 64 GB of memory. Our existing FWI code can readily achieve this for 3D models defined on a 500-μm grid which is adequate for inversion of signals to frequencies up to 1 MHz. We can include both anisotropy and attenuation in the forward modelling within that memory envelope if required. Our code is highly parallelised across multiple cores on each compute node, and across multiple compute nodes each of which can invert one or more sources simultaneously.

For the 3D FWI results that we show in the paper, we used about 100 sources per iteration. On each GPU server, we would invert one source on each of the eight GPU's, each of which has 64 GB of available memory. Across twelve such servers, we can therefore invert 96 sources simultaneously, storing everything in local GPU memory, with no inter-GPU communications, and with the only significant data transfer to/from the GPUs being the observed data which is passed once, and the updated model and two other entities (the local gradient and local hessian) that are the same size as the model; these are passed once per iteration. This data volume is straightforward to pass over InfiniBand on these machines. Consequently, the GPUs never encounter significant waits for external events, and the data, wavefields and models required during each iteration are readily stared within local GPU memory.

The heart of an FWI code is the numerical wave propagator, typically running on a regular cubic grid, employing high-order time-stepping finite-differences. More than 95% of the compute required for an FWI inversion is spent propagating waves through a model using that propagator, and almost the remainder is spend cross-correlating the forward and backward wavefields. Both these tasks are highly parallelised in existing FWI codes. Provided that we have sufficient memory and large on-board cache, these codes typically achieve better than 70% utilisation of the maximum theoretically available FLOPS. In the configuration that we suggest, both these conditions are met, and the scaling from CPU-based to GPU-based codes appears reasonable.

The capital cost of this system is about $1.2 million, and perhaps $2 million once it is powered, cooled and configured. Scaling of run times in the way we have suggested then gives a run time of about ten minutes for full 3D FWI to 850 kHz, and the amortised capital cost required to achieve that over three years is about $15 per FWI-run provided that the hardware is utilised, on average, about three times an hour to generate about 26,000 images per year. In practice, a time-critical medical compute centre will not ever want to run at 100% saturation in order to be sure that it always has capacity available to meet demand, so that the true amortised capital cost is consequently likely to be a few tens of dollars per FWI job.

SUPPLEMENTARY REFERENCES

42. Li, C., Sandhu, G. Y., Boone, M. & Duric, N. Breast imaging using waveform attenuation tomography. *SPIE Proceedings* **10139,** Ultrasonic Imaging and Tomography, 11-16 Feb 2017, Orlando, FL 2017:

43. Vigh, D., Jiao, K., Watts, D. & Sun, D. Elastic full-waveform inversion application using multicomponent measurements of seismic data collection. *Geophysics* **79,** 1MA-Z52 (2014).

44. Abe, K., Taira, T. Focused Ultrasound Treatment, Present and Future. *Neurologia medico-chirurgica* **57,** 386-391 (2017).

45. Lindsey, B. D., Light, E. D., Nicoletto, H. A., Bennett, E. R., Laskowitz, D. T., Smith, S. W. The ultrasound brain helmet: New transducers and volume registration for in vivo simultaneous multi-transducer 3-D transcranial imaging. *IEEE Transactions on Ultrasonics, Ferroelectrics, and Frequency Control,* **58,** 1189-1202 (2011).

SUPPLEMENTARY TABLE

MIDA number	speed (m/s)	density (kg/m^3)	quality factor	tissue type
1	1600.0	1174.0	156	Dura
2	1511.0	1045.0	523	Cerebellum Grey Matter
3	1510.0	1053.0	135	Pineal Body
4	1505.0	1044.0	1745	Amygdala
5	1515.0	1044.5	1745	Hippocampus
6	1504.5	1007.0	20882	CSF Ventricles
7	1510.0	1044.5	1745	Caudate Nucleus
8	1505.0	1044.5	1745	Putamen
9	1552.5	1041.5	302	Cerebellum White Matter
10	1505.0	1044.5	1745	Brain Grey Matter
11	1546.3	1045.5	299	Brainstem Midbrain
12	1552.5	1041.0	302	Brain White Matter
13	1542.0	1075.0	30	Spinal Cord
14	1546.3	1045.5	299	Brainstem Pons
15	1546.3	1045.5	176	Brainstem Medulla
16	1505.0	1044.5	1745	Nucleus Accumbens
17	1505.0	1044.5	1745	Globus Pallidus
18	1629.5	1075.0	146	Optic Tract
19	1515.0	1053.0	135	Hypophysis or Pituitary Gland
20	1505.0	1044.5	1745	Mammillary Body
21	1520.0	1044.5	1745	Hypothalamus
22	1552.5	1041.0	302	Commissura (Anterior)
23	1552.5	1041.0	302	Commissura (Posterior)
24	1578.2	1049.8	841	Blood Arteries
25	1578.2	1049.8	841	Blood Veins
26	343.0	1.2	233990	Air Internal - Ethmoidal Sinus

27	343.0	1.2	233990	Air Internal - Frontal Sinus
28	343.0	1.2	233990	Air Internal - Maxillary Sinus
29	343.0	1.2	233990	Air Internal - Sphenoidal Sinus
30	343.0	1.2	233990	Air Internal - Mastoid
31	343.0	1.2	233990	Air Internal - Nasal/Pharynx
32	1504.5	1007.0	20882	CSF General
33	1504.5	1007.0	20882	Ear Cochlea
34	1504.5	1007.0	20882	Ear Semi-circular Canals
35	1639.6	1099.5	4358	Ear Auricular Cartilage (Pinna)
36	3514.9	1908.0	16	Mandible
37	1620.7	1102.0	16	Mucosa
38	1588.4	1090.4	278	Muscle (General)
39	1639.6	1099.5	4358	Nasal Septum (Cartilage)
40	2813.7	1908.0	20	Skull (General)
41	4565.9	2180.0	11	Teeth
42	1588.4	1090.4	682	Tongue
43	1440.2	911.0	501	Adipose Tissue
44	3514.9	1908.0	16	Vertebra - C1 (atlas)
45	3514.9	1908.0	16	Vertebra - C2 (axis)
46	3514.9	1908.0	16	Vertebra - C3
47	3514.9	1908.0	16	Vertebra - C4
48	3514.9	1908.0	16	Vertebra - C5
49	1639.6	1099.5	4358	Intervertebral Discs
50	1480.0	1000.0	20000	Background
51	1624.0	1109.0	91	Epidermis/Dermis
52	2117.5	1178.3	32	Skull - Diploë
53	2813.7	1908.0	20	Skull - Inner Table
54	2813.7	1908.0	20	Skull - Outer Table
55	1643.3	1075.5	2868	Eye Lens
56	1638.8	1032.0	1633	Eye Retina/Choroid/Sclera
57	1525.8	1004.5	412	Eye Vitreous
58	1562.5	1050.5	5464	Eye Cornea
59	1525.8	1004.5	412	Eye Aqueous
60	1588.4	1090.4	278	Muscle - Platysma
61	1750.0	1142.0	124	Tendon - Galea Aponeurotica
62	1477.0	911.0	304	Subcutaneous Adipose Tissue
63	1588.4	1090.4	278	Muscle - Temporalis/Temporoparietalis
64	1588.4	1090.4	278	Muscle - Occipitiofrontalis - Frontal
65	1588.4	1090.4	278	Muscle - Lateral Pterygoid
66	1588.4	1090.4	278	Muscle - Masseter
67	1588.4	1090.4	278	Muscle - Splenius Capitis
68	1588.4	1090.4	278	Muscle - Sternocleidomastoid
69	1588.4	1090.4	278	Muscle - Occipitiofrontalis - Occipital
70	1588.4	1090.4	278	Muscle - Trapezius
71	1588.4	1090.4	278	Muscle - Mentalis
72	1588.4	1090.4	278	Muscle - Depressor Anguli Oris
73	1588.4	1090.4	278	Muscle - Depressor Labii
74	1588.4	1090.4	278	Muscle - Nasalis
75	1588.4	1090.4	278	Muscle - Orbicularis Oris

76	1588.4	1090.4	278	Muscles - Procerus
77	1588.4	1090.4	278	Muscle - Levator Labii Superioris
78	1588.4	1090.4	278	Muscle - Zygomaticus Major
79	1588.4	1090.4	278	Muscle - Orbicularis Oculi
80	1588.4	1090.4	278	Muscle - Levator Scapulae
81	1588.4	1090.4	278	Muscle - Medial Pterygoid
82	1588.4	1090.4	278	Muscle - Zygomaticus Minor
83	1588.4	1090.4	278	Muscles - Risorius
84	1588.4	1090.4	278	Muscle - Buccinator
85	343.0	1.2	233990	Ear Auditory Canal
86	343.0	1.2	233990	Ear Pharyngotympanic Tube
87	2117.5	1178.3	32	Hyoid Bone
88	1559.5	1048.0	216	Submandibular Gland
89	1559.5	1048.0	216	Parotid Gland
90	1559.5	1048.0	216	Sublingual Gland
91	1588.4	1090.4	278	Muscle - Superior Rectus
92	1588.4	1090.4	278	Muscle - Medial Rectus
93	1588.4	1090.4	278	Muscle - Lateral Rectus
94	1588.4	1090.4	278	Muscle - Inferior Rectus
95	1588.4	1090.4	278	Muscle - Superior Oblique
96	1588.4	1090.4	278	Muscle - Inferior Oblique
97	343.0	1.2	233990	Air Internal - Oral Cavity
98	1750.0	1142.0	124	Tendon - Temporalis Tendon
99	1505.0	1044.5	1745	Substantia Nigra
100	1552.5	1041.0	302	Cerebral Peduncles
101	1629.5	1075.0	146	Optic Chiasm
102	1629.5	1075.0	146	Cranial Nerve I - Olfactory
103	1629.5	1075.0	146	Cranial Nerve II - Optic
104	1629.5	1075.0	146	Cranial Nerve III - Oculomotor
105	1629.5	1075.0	146	Cranial Nerve IV - Trochlear
106	1629.5	1075.0	146	Cranial Nerve V - Trigeminal
107	1629.5	1075.0	146	Cranial Nerve V2 - Maxillary Division
108	1629.5	1075.0	146	Cranial Nerve V3 - Mandibular Division
109	1629.5	1075.0	146	Cranial Nerve VI - Abducens
110	1629.5	1075.0	146	Cranial Nerve VII - Facial
111	1629.5	1075.0	146	Cranial Nerve VIII - Vestibulocochlear
112	1629.5	1075.0	146	Cranial Nerve IX - Glossopharyngeal
113	1629.5	1075.0	146	Cranial Nerve X - Vagus
114	1629.5	1075.0	146	Cranial Nerve XI - Accessory
115	1629.5	1075.0	146	Cranial Nerve XII - Hypoglossal
116	1505.0	1044.5	1745	Thalamus

Supplementary Table 1. Physical properties of tissue types. Tissue types in the MIDA model [40] together with their corresponding acoustic wave speeds, densities and anelastic absorptions (expressed as quality factor *Q*) used to generate the true model, taken from [24-27, 29-34]. The properties of most minor tissue types are estimates. Some properties of major tissue types are not well characterized by available experimental measurements; values of anelastic absorption are especially difficult to measure in the laboratory. Provided that these estimates are broadly correct, their exact values do not influence the quality of the recovered images.
